# Are the 10 Meter and 6 Minute Walk Tests Redundant in Patients with Spinal Cord Injury?

**DOI:** 10.1371/journal.pone.0094108

**Published:** 2014-05-01

**Authors:** Gail F. Forrest, Karen Hutchinson, Douglas J. Lorenz, Jeffrey J. Buehner, Leslie R. VanHiel, Sue Ann Sisto, D. Michele Basso

**Affiliations:** 1 Human Performance and Engineering Laboratory, Kessler Foundation Research Center, West Orange, New Jersey, United States of America; 2 Department of Physical Medicine and Rehabilitation, Rutgers, New Jersey Medical School, Newark, New Jersey, United States of America; 3 Department of Physical Therapy and Athletic Training, Boston University, Boston, Massachusetts, United States of America; 4 Department of Bioinformatics and Biostatistics, School of Public Health and Information Sciences, University of Louisville, Louisville, Kentucky, United States of America; 5 Kentucky Spinal Cord Research Center, University of Louisville, Louisville, Kentucky, United States of America; 6 Wexner Medical Center at the Ohio State University- Dodd Hall, Columbus, Ohio, United States of America; 7 Hulse Spinal Cord Injury Lab and Crawford Research Institute, Shepherd Center, Atlanta, Georgia, United States of America; 8 State University of New York at Stony Brook, School of Health Technology and Management, Research and Development Park, Rehabilitation Research and Movement Performance Laboratory, Stony Brook, New York, United States of America; 9 The Ohio State University, School of Allied Medical Professions, Center for Brain and Spinal Cord Repair, Columbus, Ohio, United States of America; University of Toronto, Canada

## Abstract

**Objective:**

To evaluate the relationship and redundancy between gait speeds measured by the 10 Meter Walk Test (10MWT) and 6 Minute Walk Test (6MWT) after motor incomplete spinal cord injury (iSCI). To identify gait speed thresholds supporting functional ambulation as measured with the Spinal Cord Injury Functional Ambulation Inventory (SCI-FAI).

**Design:**

Prospective observational cohort.

**Setting:**

Seven outpatient rehabilitation centers from the Christopher and Dana Reeve Foundation NeuroRecovery Network (NRN).

**Participants:**

249 NRN patients with American Spinal Injury Association Impairment Scale (AIS) level C (n = 20), D (n = 179) and (n = 50) iSCI not AIS evaluated, from February 2008 through April 2011.

**Interventions:**

Locomotor training using body weight support and walking on a treadmill, overground and home/community practice.

**Main Outcome Measure(s):**

10MWT and 6MWT collected at enrollment, approximately every 20 sessions, and upon discharge.

**Results:**

The 10MWT and 6MWT speeds were highly correlated and the 10MWT speeds were generally faster. However, the predicted 6MWT gait speed from the 10MWT, revealed increasing error with increased gait speed. Regression lines remained significantly different from lines of agreement, when the group was divided into fast (≥0.44 m/s) and slow walkers (<0.44 m/s). Significant differences between 6MWT and 10MWT gait speeds were observed across SCI-FAI walking mobility categories (Wilcoxon sign rank test p<.001), and mean speed thresholds for limited community ambulation differed for each measure. The smallest real difference for the 6MWT and 10MWT, as well as the minimally clinically important difference (MCID) values, were also distinct for the two tests.

**Conclusions:**

While the speeds were correlated between the 6MWT and 10MWT, redundancy in the tests using predictive modeling was not observed. Different speed thresholds and separate MCIDs were defined for community ambulation for each test.

## Introduction

In people with incomplete spinal cord injury (iSCI), walking capacity - comprised of walking speed and endurance - is an important construct in evaluating efficacy of gait training rehabilitation[1]. Currently, the most accepted standardized timed tests used in studies of persons with iSCI are the 10 Meter Walk Test (10MWT) for speed and 6 Minute Walk Test (6MWT) for walking endurance[2]. These outcome measures are valid, reliable and responsive for acute to chronic iSCI[3]. The pragmatic characteristics of timed walking tests, such as ease and time burden to perform, often determine whether both will be used for clinical outcomes or research trials.

Recent studies advocate the use of only a single walking test, specifically the 10MWT, for clinical research in individuals with SCI[4]; [5] due to ease of administration. When compared to age-matched normative data[6] and data on stroke survivors after rehabilitation[7], the 10MWT and 6MWT showed little clinical differences for speed. Additionally, strong correlations between the 10MWT and the 6MWT exist at a single time point after recovery periods for individuals with iSCI[8]; [9]. However, each walking test appears to perform differently when measuring preferred vs. maximum walking speed. Higher speeds occurred most often with the 10MWT compared to the 6MWT when measuring maximum speeds, but during tests of preferred speed, the highest speeds occurred most often using the 6MWT[5]; [10]. Thus, measured walking speed appears to differ for the two walking tests according to walking speed demands. Indeed, Barbeau[4] found that people with iSCI produced similar walking speeds on the 15.2 Meter Walking Test (15.2 MWT) and the 6MWT when maximum speeds were below 0.9 m/s. However, when higher speeds were attained, the two walking tests produced significantly different values from each other. These differences were postulated to be *clinically* irrelevant, because subjects were all independent ambulators and considered to have sufficient capacity to perform unrestricted community walking, although the community ambulation scores were not reported. Thus, there is a need to determine whether different walking speeds on the two tests reflect true differences in function and community ambulation. Furthermore, although speed thresholds for community ambulation after iSCI been described for the 10MWT[11] they have yet to be identified for the 6MWT.

Support for the use of a single walking test may lie in the interpretation of the redundancy between walking tests. Barbeau et al. considered redundancy to be the degree of equivalency, or the comparison between the average gait speed during a short walking test and the 6MWT[4]. Another method for evaluating redundancy is to model the relationship between the 10MWT and the 6MWT and compare the predicted 6MWT walking speed (from the 10MWT data) to the actual 6MWT speed. The two walking tests would be considered redundant if the error between the predicted and actual values was small across all ranges of gait speed. Thus, knowledge of speed on one test could be used to accurately predict speed on the other, mitigating the need for conducting both tests. To our knowledge, predictive modeling has not been used to determine if the 10MWT and 6MWT provide unique measures of walking capacity across a range of speeds and over time after iSCI.

To delineate the unique or redundant contributions of the 10MWT and the 6MWT, a measure of functional capacity must be used. Recently gait speed from the 10MWT was validated as a predictor of community ambulation in a large European study[11]. A minimum gait speed of 0.44 m/s was the threshold for community ambulation as determined by partitioning components of the Spinal Cord Independence Measure (SCIM)[11]. By contrast, the mobility portion of the Spinal Cord Injury Functional Ambulation Inventory (SCI-FAI), which relies on patient self-report of gait parameters and frequency of walking in the home and community, has not been examined relative to gait speed. The SCI-FAI is a reliable, valid and sensitive measure of walking ability in individuals with SCI[12]. In the present study, we analyzed gait speed at each successive level of ambulatory capacity, as defined by the SCI-FAI mobility scale, to determine the validity of walking speed measurements in discriminating household and community ambulation. We also separately analyzed the 6MWT and 10MWT data using the 0.44 m/s threshold for identifying slow (household) and fast (community) gait speeds[11].

The first purpose of this study was to evaluate the relationship and redundancy between gait speeds measured by the 10MWT and the 6MWT after motor iSCI. If redundancy exists then the scores should be highly correlated and one of the measures would predict performance of the other. Speeds collected before, during and after Locomotor Training rehabilitation will determine whether differences between the two walk tests depend on the extent of recovery. The second purpose was to examine if the variability between these gait speed measures was unique at fast or relatively slow gaits speeds. We propose that there are differences in walking test performance, not previously identified, for both slow walking speeds (less than 0.44 m/s) and fast walking speeds (those equal or greater than 0.44 m/s)[13]. The third purpose was to establish whether statistical and clinically relevant differences exist between the 10MWT and 6MWT. Measures of smallest real difference (SRD) and functional walking capacity (e.g. household vs. community ambulation with SCI-FAI) were examined. We propose that differences in the inter-evaluation variability between the walking tests may best signify whether both tests are clinically meaningful, or if only one test is needed.

## Methods

### Study Participants

Two hundred forty-nine patients, enrolled in the standardized Locomotor Training therapy program described in detail elsewhere[14]–[16]. Patients who were admitted between February 2008 through April 2011 were evaluated at seven out-patient clinical sites in the Christopher and Dana Reeve Foundation (CDRF) NeuroRecovery Network (NRN). Sites included Boston Medical Center, Boston, MA; Frazier Rehabilitation Institute, Louisville, KY; Kessler Institute for Rehabilitation, West Orange, NJ; Magee Rehabilitation Hospital, Philadelphia, PA; the Ohio State University Medical Center, Columbus, OH; Shepherd Center, Atlanta, GA; and The Institute for Rehabilitation and Research, Houston, TX. From each center an IRB-approved written statement of consent was obtained in writing prior to collecting clinical information and administering the outcome measures. Participants provided their written informed consent to participate in this study. The IRB institutions were as follows: Institutional Review Board of Boston University Medical Campus and Boston Medical Center, Boston, MA; Kentucky One Health Research Center, Institute of Review Board, Louisville, KY; Kessler Foundation Institutional Review Board, West Orange, NJ; Magee Rehabilitation Institutional Review Board, Philadelphia, PA; Biomedical Sciences Institutional Review Board, the Ohio State University, Columbus, OH; Research Review Committee at Shepherd Center, Atlanta, GA; University of Texas Health Science Center Houston, Texas. Patients were selected for participation in the NRN Locomotor Training program and outcome assessments based on 1) the presence of a non-progressive spinal cord lesion, 2) neurological level of injury above T11 as determined by the International Standards for Neurological Classification of Spinal Cord Injury (ISNCSCI), 3) completion of an in-patient rehabilitation program, 4) no use of Botox or other medications for chemodenervation for spasticity for the 3 months prior to enrollment, 5) some lower limb movement or visible voluntary contraction, 6) the capacity to generate a lower limb reciprocal alternating flexion/extension stepping pattern in the body-weight supported step training environment, and 7) medical referral by a physician for physical therapy. Patients on anti-spasticity medications were weaned during participation in the NRN program as directed by their NRN physicians. The patients underwent at least the baseline evaluation, a minimum of 20 training sessions and at least one additional evaluation of the functional outcome measures.

### Outcome Measures

The 6MWT and the 10MWT were captured as part of a battery of measures at baseline in a single session or over two consecutive days, depending on the abilities of the participants to perform them. These measures were captured approximately every 20 treatment sessions thereafter, and at discharge from the Locomotor Training program. The standardized procedures for gait assessment within the NRN have been outlined in a previous manuscript[16]. For the 6MWT, the placement of turns, precise verbal feedback and location of the observer conformed to standardized methods[17]. The need for physical assistance or to sit ended the test. For the 10MWT, a 14-meter path with a flying start was used to avoid acceleration/deceleration effects associated with starting and stopping during this assessment. The middle 10 meters of this path were used for the measurement. Patients were instructed to “walk as fast as they can”. Both tests included use of assistive devices when required; however, no lower limb bracing or physical assistance was allowed. When patients changed assistive devices over the course of treatment, each gait outcome measure was conducted twice – once with the device used at enrollment, termed the “initial device” and once with the device currently being used, termed the “current device”. Five minutes of seated rest preceded each of the gait tests. Our data represent the fastest walking speed attained, irrespective of ambulation device. The walking mobility scale of the SCI-FAI was used to classify the individuals’ self- reported level of home or community ambulation. The walking mobility portion of the SCI-FAI scale classifies individuals from 0 to 5. A score of 0 indicates self-reported non-ambulatory status or ambulation with physical assistance only; scores 1–3 indicate in-home but not community ambulation; and scores 4–5 indicate limited and independent community ambulation ability.

### Data Analysis

Of 249 patients, 6MWT and 10 MWT data were available for 217 at enrollment, 249 at discharge and 249 at interim evaluations. Thirty-two (32) were unable to complete one or both of the walk tests at enrollment. One hundred seventy patients had enrollment and discharge measurements of the 10MWT, 6MWT and SCI-FAI mobility measure. On this sample, we calculated the SRDs and conducted the analysis of changes in the 10MWT and 6MWT. One hundred twenty-five patients had enrollment and discharge measurements of the 10MWT and 6MWT as well as an enrollment SCI-FAI score below 5. On this sample, we calculated minimum clinically important differences (MCIDs). The details of the calculation of SRD and MCID are provided below.

The relationship between the 10MWT and 6MWT was evaluated using correlation and regression methods for measurements taken at enrollment, discharge, and over the entire period of participation in the NRN. Simple linear regression models were fit and Pearson correlation coefficients calculated on the enrollment and discharge data using 10MWT speed as the predictor and 6MWT speed as the outcome. The regression models were fit with generalized least squares to permit the modeling of heterogeneous residual variance. The same models – 6MWT speeds predicted by 10MWT speeds – were fit for all of the data (i.e. enrollment, interim, and discharge measurements) using the linear mixed effects model. The 10MWT speed served as the only fixed and random effect, and variance functions to model heterogeneous variance patterns were included[18]. Pearson correlation coefficients calculated on the full data were calculated utilizing recently developed methods for clustered data[19]. We calculated these clustered data coefficients to account for dependence among repeated evaluations of NRN patients and control for the potential biasing effect of informative cluster size, in this case the varying number of observations contributed by NRN patients.

We compared 10MWT and 6MWT speeds over categories defined by the SCI-FAI mobility subscale using the Kruskal-Wallis test, with pairwise comparisons of SCI-FAI categories conducted using the Wilcoxon rank sum test with the Hochberg correction for multiple testing[20]. We compared the measurement properties of the 10MWT and 6MWT by calculating the SRD and the MCID. The SRD was calculated according to a modified version of a formula proposed by Beckerman[21]; a discussion of the modification to the SRD formula is in the Appendix (Appendix S1). The MCID was calculated through a receiver-operator characteristic (ROC) analysis utilized by Tilson, et al.[22] which we describe briefly. We selected a one-unit increase in the SCI-FAI mobility subscale to represent clinically relevant change in a patient’s walking function and divided patients into responders and non-responders based on this criterion[12]; [22]. We constructed ROC curves for enrollment-to-discharge changes in the 10MWT and 6MWT, from which we calculated the area under the curve (AUC) with 95% confidence intervals. The walk tests were defined to be in significant correspondence with 1-unit increases in the SCI-FAI if the 95% confidence interval for the AUC did not contain 0.5. Each point on the ROC curve defines a threshold for the change in walking speed, above which a patient is classified as a responder and below a non-responder. At each of these speed thresholds on the ROC curve we calculated two quantities: (1) sensitivity, defined as the proportion of patients classified as responders among those experiencing clinically relevant improvement (a one-unit increase in SCI-FAI), and (2) specificity, defined as the proportion of patients classified as non-responders among those not experiencing clinically relevant improvement. We defined the MCID to be the threshold at which the sum of the sensitivity and specificity was maximized[23]. All analyses were conducted using the full data and for two subgroups of data – slow walks, defined as walk speeds less than 0.44 m/s, and fast walks were equal to or greater than 0.44 m/s per van Hedel, 2009[11]; [23]. Demographic and clinical characteristics at NRN enrollment were summarized using means and standard deviations for continuous, symmetric data, medians and extrema for continuous skewed data, and counts and percentages for categorical data. Hypothesis tests were conducted at the .05 significance level. Analyses were conducted using the open-source R software package[24].

## Results

### Demographic, Clinical, and Treatment Characteristics

The demographic and clinical characteristics of our sample of 249 patients (Table 1) corresponded with those of other samples of NRN data[8]; [13]; [25] and with the incomplete SCI population[26]. Patients were enrolled in the NRN for a median 3.4 months and received a median of 40 treatment sessions, with the highest enrollment time and number of completed treatment sessions being 52.5 months and 353 sessions, respectively. The number of evaluations ranged from 2–18 with a median of 4 evaluations per patient at which 6MWT and 10MWT were measured.

**Table 1:** Demographic, clinical, and treatment characteristics.

Demographics (N = 249)	
Sex	
F	59 (24)
M	190 (76)
Age	42±16
AIS	
C	20 (8)
D	179 (72)
* Not evaluated*	*50 (20)*
Time Since SCI (years)	0.7 [0.1, 21.6]
Mechanism of Injury	
MVA	83 (33)
Fall	54 (22)
Sporting	45 (18)
Med-Surg	25 (10)
Violence	17 (7)
Non-Trauma	15 (6)
Other	10 (5)
**Treatment Characteristics**	
Time in NRN (months)	3.4 [0.2, 52.5]
Treatment Sessions	40 [2, 353]
Evaluations	4 [2], [18]

Values are counts (percentages), mean ± SD, or median [min, max].

### Little Redundancy between 10MWT and 6MWT Gait Speeds

**Figure 1:**
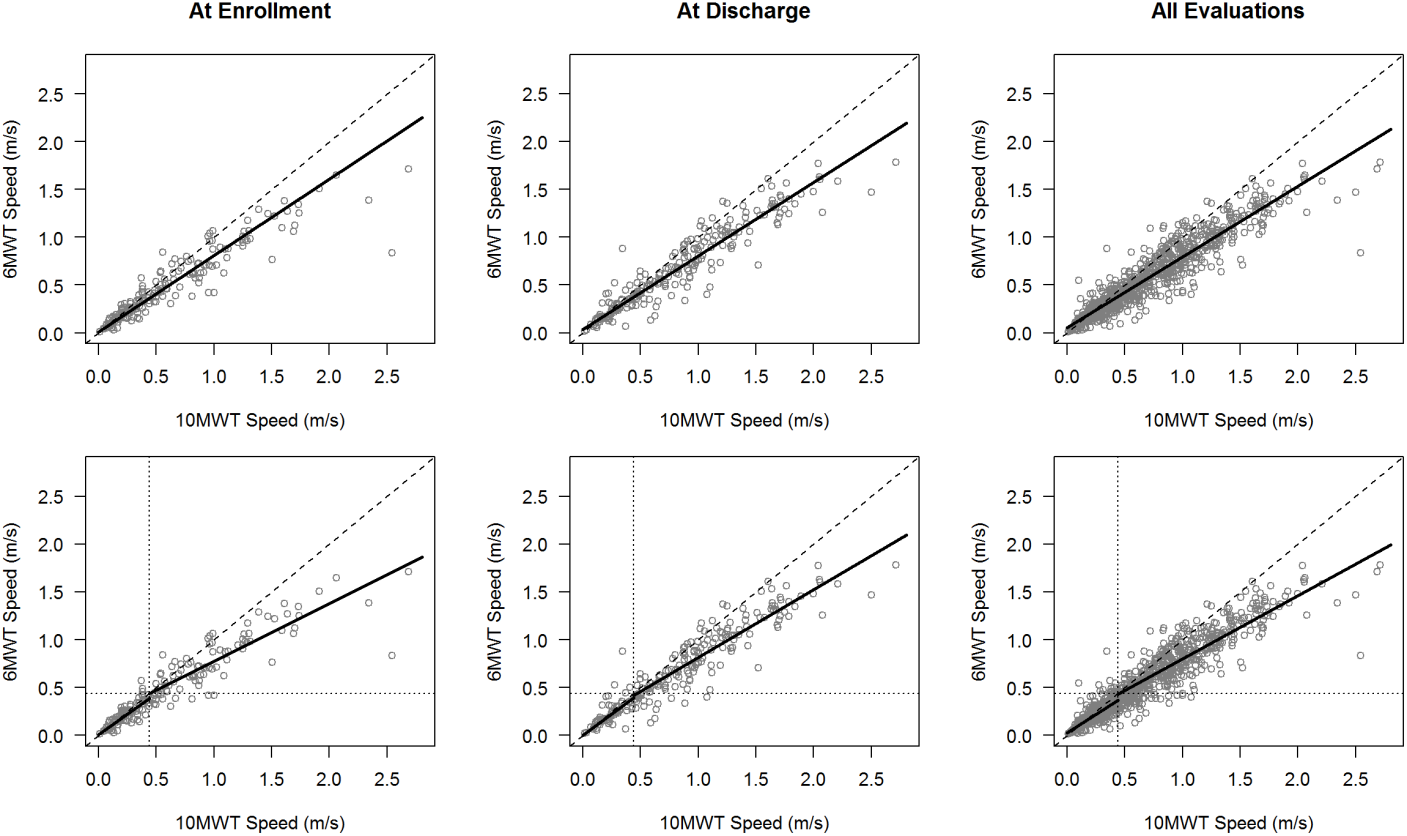
Lines of best fit from linear models predicting 6MWT speeds with 10MWT speeds at enrollment, discharge, and all evaluations. Top row: linear models fit to all observations. Bottom row: linear models fit to slow and fast walkers (separated by dotted line). Dashed line is 45-degree line of agreement.

To examine redundancy, we reasoned that the scores for the 10MWT and the 6MWT should be highly correlated and one measure would predict the performance on the other. Speeds from the 10MWT and 6MWT were highly correlated (Table 2, Figure 1) at enrollment (0.93), at discharge (0.94) and for all evaluations (r = 0.94). Speeds from the 10MWT were generally faster than those from the 6MWT. However, it is noteworthy that for up to 23% of cases, gait speeds on 6MWT were faster than 10MWT. Corresponding to each plot, in Figure 1 we compared the regression line of best fit with the line of agreement, defined by an intercept of 0 and a slope of 1, as a measure of redundancy. The lines differed significantly at enrollment, at discharge and for all evaluations, indicating that speeds from the 10MWT and 6MWT were not equivalent (F-test, p<.001; Figure 1).

**Table 2:** Assessment of linear relationship between 6MWT and 10MWT via correlation and regression.

Walk Type	Evaluations	Correlation	Intercept	Slope
All Walks	Enrollment (N = 217)	0.93 (0.91, 0.95)	0.01 (0.00, 0.02)	0.80 (0.76, 0.84)
	Discharge (N = 240)	0.94 (0.92, 0.95)	0.04 (0.01, 0.06)	0.77 (0.74, 0.80)
	All (N = 249, 1028 observations)	0.94 (0.92, 0.96)	0.05 (0.04, 0.07)	0.74 (0.71, 0.77)
Slow Walks	Enrollment (N = 123)	0.84 (0.76, 0.89)	0.01 (0.00, 0.01)	0.87 (0.79, 0.94)
	Discharge (N = 68)	0.73 (0.59, 0.83)	0.00 (−0.01, 0.02)	0.89 (0.79, 0.99)
	All (N = 143, 469 observations)	0.80 (0.73, 0.86)	0.03 (0.01, 0.04)	0.77 (0.71, 0.84)
Fast Walks	Enrollment (N = 94)	0.85 (0.78, 0.90)	0.17 (0.10, 0.24)	0.60 (0.52, 0.68)
	Discharge (N = 172)	0.90 (0.86, 0.92)	0.10 (0.04, 0.16)	0.71 (0.66, 0.76)
	All (N = 178, 559 observations)	0.89 (0.85, 0.92)	0.14 (0.10, 0.18)	0.66 (0.62, 0.70)

For enrollment, discharge, and all evaluations, nonparametric Spearman correlation coefficients and slopes and intercepts from lines of best fit are given with 95% confidence intervals. Results are presented for all walk evaluations, slow walk evaluations (<.44 m/s) and fast walk evaluations (≥.44 m/s).

**Figure 2:**
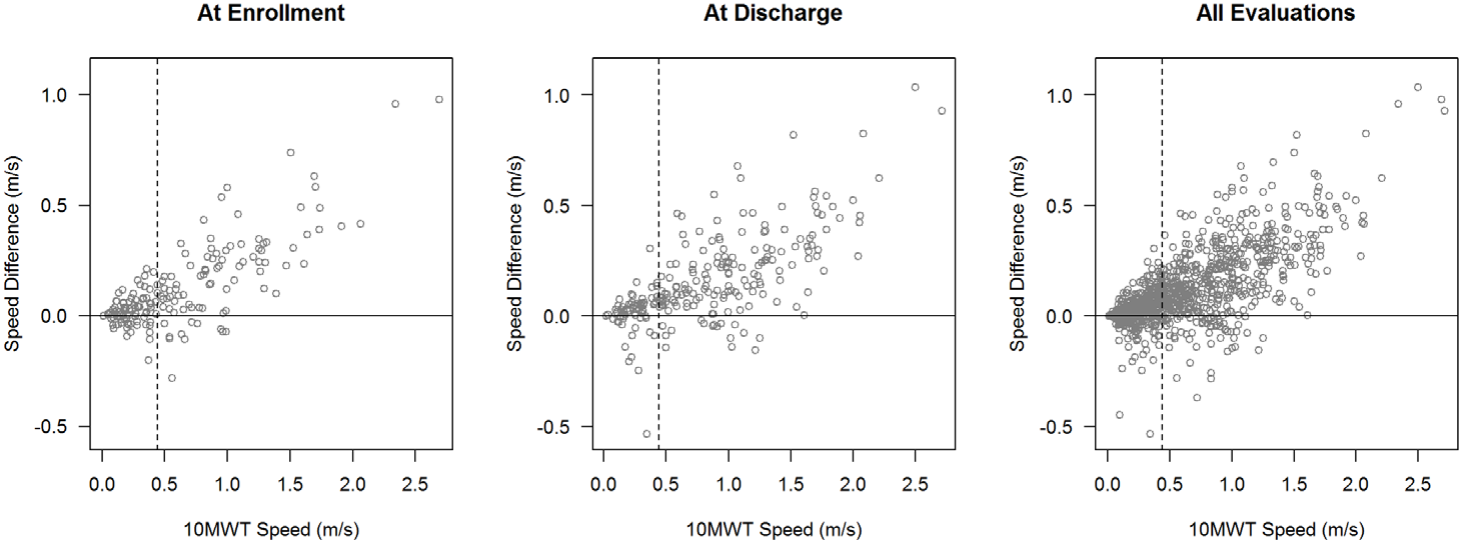
Difference in walk speeds (10MWT−6MWT) by speed on 10MWT for enrollment, discharge, and all evaluations. Vertical dashed line is at 0.44/s, separating slow and fast walkers. Negative values represent faster gait speeds on the 6MWT than the 10MWT.

From the linear models, we predicted the 6MWT gait speed from 10MWT and examined the error in prediction across a range of speeds (Table 3, Figure 2). In fitting the linear mixed effects models, we modeled the residual variance as an increasing power function of 10MWT speed (see the Appendix S1, for technical specifications and details of modeling variance heterogeneity). At the enrollment evaluation, residual standard error increased from 0.05 m/s to 0.31 m/s as 6MWT speeds increased from 0.20 m/s to 2.0 m/s. Comparable increases were observed for discharge evaluations (error increased from 0.08 m/s to 0.21 m/s) and for all evaluations (0.07 m/s to 0.22 m/s).

**Table 3:** Residual standard errors for linear models using 10MWT speeds to predict 6MWT speeds at selected speeds.

10MWT Speed (m/s)	FULL MODEL			SLOW/FAST MODELS		
	Enrollment(N = 217)	Discharge(N = 240)	All(N = 249)	Enrollment (N = 217)	Discharge(N = 240)	All(N = 249)
0.2	0.05	0.08	0.07	0.05	0.06	0.07
0.4	0.08	0.11	0.10	0.09	0.11	0.10
0.6	0.12	0.13	0.12	0.12	0.15	0.14
0.8	0.15	0.15	0.14	0.14	0.16	0.15
1.0	0.18	0.16	0.15	0.16	0.16	0.16
1.25	0.21	0.18	0.17	0.19	0.17	0.17
1.5	0.25	0.19	0.19	0.21	0.17	0.17
2.0	0.31	0.21	0.22	0.25	0.18	0.18

Standard errors were modeled as a power function of the 10MWT speeds. Results presented for modeling all of the data and for modeling slow and fast walkers separately. Models including the heterogeneous variance function fit the data significantly better than models without (ANOVA F-test, p<.001), justifying their use in models of the 6MWT and 10MWT.

### Inequalities between 10MWT and 6MWT Occur for Fast and Slow Walkers

**Figure 3:**
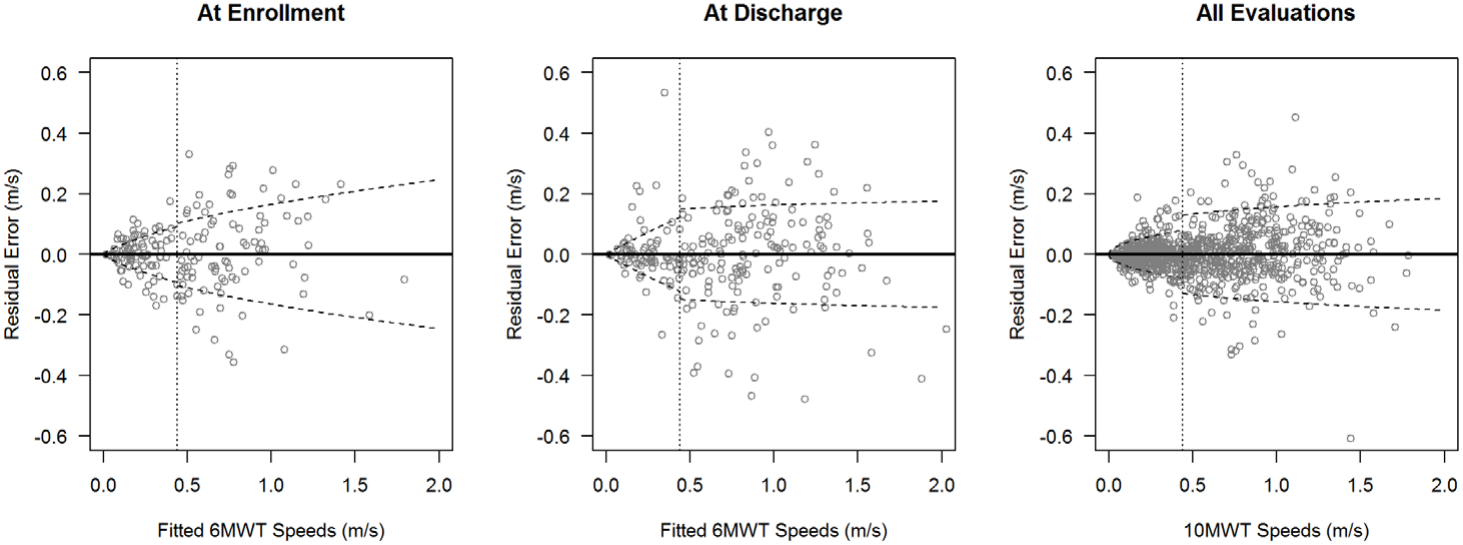
Residuals from linear models predicting 6MWT speeds with 10MWT speeds at enrollment, discharge, and all evaluations. Linear models fit to slow and fast walkers (separated by dotted line). Dashed lines are estimated residual standard errors from models.

Given that Barbeau et al.[4] established differences at faster speeds and we established substantial error at slow speeds we repeated the analyses of the relationship between the 10MWT and 6MWT for two groups of patients – fast walkers, those with gait speeds meeting or exceeding 0.44 m/s, and slow walkers, with gait speeds less than 0.44 m/s. This cutoff was selected based on prior research identifying 0.44 m/s as a threshold for community ambulation[13]; [27]. Compared to the overall sample, correlations between the 10MWT and 6MWT were reduced within the fast group and, to a greater extent, the slow group (Table 2, Figure). Gait speeds from the 10MWT continued to exceed 6MWT speeds in the two groups, although to a lesser extent in the slow walk group (Figure 3). Sixty-six percent (55 of 83) of enrollment evaluations, 73% (46/63) of discharge evaluations and 70% (282/404) of all evaluations had 10MWT speeds greater than 6MWT speeds for slow walkers. Conversely, up to 34% of gait speeds were higher during the 6MWT for slow walkers. For fast walkers, 85% (80/94) of enrollment evaluations, 88% (152/172) of discharge evaluations and 86% (480/559) of all evaluations registered higher speeds on the 10MWT than the 6MWT. Conversely, up to 15% of gait speeds were higher during the 6MWT for fast walkers. The lines of best fit remained significantly different from the lines of agreement in both slow and fast walking groups (Figure 1, Table 2, p<.001), although disparity from the line of agreement was greater in the fast group. The slopes of the regression lines for fast walkers were substantially below the line of agreement for each plot while the slopes for slow walkers were modestly displaced. Linear models fit to the two subgroups continued to exhibit increasing prediction errors with increased gait speed (Table 3, Figure 3), which was modeled with a power function as before.

### Smallest Real Difference is Lower for 6MWT

**Figure 4:**
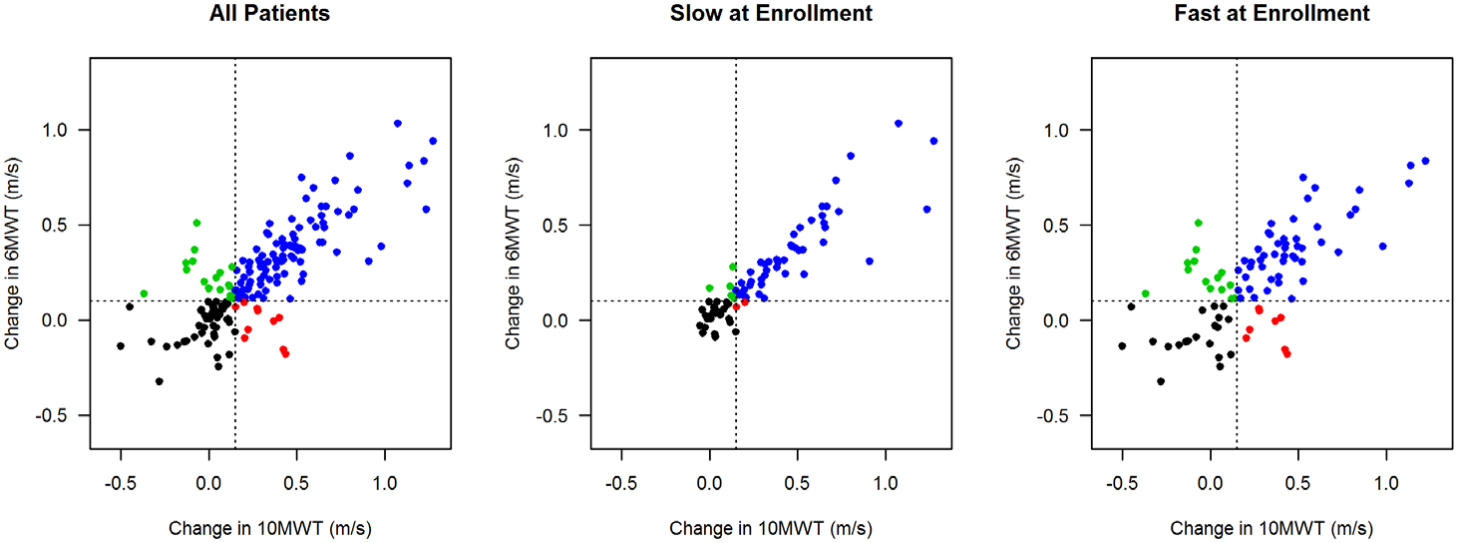
Enrollment-to-discharge improvements in 6MWT speed by enrollment-to-discharge improvements in 10MWT speed for all patients (n = 170), patients that walked slow (<0.44 m/s) at enrollment (n = 78), and patients that walked fast (≥0.44 m/s) at enrollment (n = 92). Plots restricted to patients that had enrollment and discharge evaluations of the 6MWT and 10MWT. Dotted lines are plotted at the MCID for the 6MWT (0.10 m/s) and 10MWT (0.15 m/s). Blue = improvements exceeded MCID for both 6MWT and 10MWT, red = improvements exceeded MCID for 10MWT only, green = improvements exceeded MCID for 6MWT only, black = improvements did not exceed MCID for either 6MWT or 10MWT.

To determine whether the differences in 6MWT and 10MWT surpassed the error of the measurement we calculated the SRD. Average improvement from enrollment to discharge in 10MWT and 6MWT speeds was 0.30 m/s and 0.26 m/s, respectively (Table 4, Figure 4), and slow walkers tended to show greater improvement than fast walkers. Previous reports[9]; [28] have estimated the test-retest intraclass correlation coefficient of both the 6MWT and 10MWT to be 0.98. Using this estimate and the estimated standard deviation of enrollment-to-discharge changes in the 6MWT and 10MWT, we calculated the SRD for the 6MWT and 10MWT to be 0.08 m/s and 0.10 m/s, respectively. The SRDs for walkers defined as slow (<0.44 m/s) and fast (≥0.44 m/s) at enrollment were nearly identical to the SRD for the overall sample (Table 4).

**Table 4:** Walking speeds at enrollment and discharge (m/s), smallest real difference (SRD), minimal clinically important difference (MCID), and area under the curve (AUC) calculated for 6MWT and 10MWT (in m/s), all and by speed group at enrollment.

Measure	6MWT			10MWT		
	All	Slow	Fast	All	Slow	Fast
**Speed at Enrollment (m/s)**	0.40±0.39	0.13±0.13	0.77±0.32	0.51±0.53	0.15±0.14	0.98±0.48
**Speed at Discharge (m/s)**	0.67±0.43	0.42±0.29	0.99±0.38	0.81±0.55	0.50±0.37	1.23±0.48
**Change in Speed (m/s)**	0.26±0.28	0.28±0.28	0.23±0.26	0.30±0.35	0.35±0.35	0.25±0.35
**SRD**	0.08	0.08	0.07	0.10	0.10	0.10
**MCID**	0.10	0.11	NA	0.15	0.15	NA
**AUC**	0.85(0.77, 0.94)	0.90(0.84, 0.96)	0.66(0.31, 1)	0.83(0.73, 0.92)	0.87(0.79, 0.94)	0.62(0.23, 1)

NA: among fast walkers, the 6MWT and 10MWT did not significantly correspond with clinically relevant change and reliable MCID could not be calculated.

### Gait Speeds and Functional Walking Capacity Classified by SCI-FAI

To understand whether clinical measures of gait speed align with functional walking, we partitioned patients with the SCI-FAI walking mobility scale into non-walkers (score 0), limited in-home ambulators (scores 1–3) and community ambulators (scores 4–5). A small number of patients (n = 19) who indicated a SCI-FAI score of 0 ambulated sufficiently at initial evaluation in the clinic to complete the 10MWT and 6MWT, albeit at gait speeds near 0 m/s (Table 5). Significantly higher speeds occurred with higher classifications for both the 6MWT and 10MWT (Kruskall-Wallis tests, p<.001, Table 5). Those classified as extensive community ambulators (SCI-FAI 5) had significantly faster gait speeds than all other classifications regardless of which walking test was used to measure gait speed (Wilcoxon test, Hochberg correction, p<.001). Gait speeds for adjacent SCI-FAI categories 1–4 were not significantly different (p>.06) whereas separations of more than one category resulted in significant differences using either walking measure (p<.04). For example, gait speeds in patients with SCI-FAI score of 2 did not significantly differ from gait speeds in patients with SCI-FAI scores 1 or 3, but were significantly lower than gait speeds of patients with SCI-FAI scores of 4 or 5.

**Table 5:** Summary statistics for 6MWT and 10MWT at enrollment, discharge, and all evaluations by SCI-FAI Walking Mobility score.

Enrollment			Discharge		
SCI-FAI	6MWT (m/s)	10MWT(m/s)	SCI-FAI	6MWT(m/s)	10MWT(m/s)
0 (N = 18)	0.01±0.03	0.02±0.04	0 (N = 3)	0.18±0.21	0.15±0.13
1 (N = 20)	0.18±0.13	0.17±0.09	1 (N = 2)	0.16±0.08	0.17±0.07
2 (N = 19)	0.27±0.26	0.26±0.21	2 (N = 7)	0.18±0.10	0.29±0.17
3 (N = 21)	0.28±0.13*	0.37±0.19	3 (N = 16)	0.28±0.20*	0.30±0.20
4 (N = 38)	0.39±0.21*	0.49±0.32	4 (N = 30)	0.44±0.28*	0.58±0.34
5 (N = 61)	0.84±0.35*	1.07±0.54	5 (N = 118)	0.89±0.37*	1.08±0.50

Values are mean ± SD. SCI-FAI score 0 indicates non-ambulatory status or ambulation with physical assistance only, scores 1–3 indicate in-home but no community ambulation, and scores 4–5 indicate occasional or regular community ambulation. Table includes 177 patients with enrollment and discharge measurements of the SCI-FAI, 6MWT, and 10MWT. SCI-FAI categories 3, 4, and 5 exhibited significant 6MW–10MW differences.

*p<.04.

### 10MWT and 6 MWT Differ from Each other across Functional Classifications

The 10MWT and 6MWT did not perform equally for each walking category on the SCI-FAI. For most SCI-FAI levels, mean gait speed on the 6MWT was slower than that on the 10MWT (Table 5). The differences between 10MWT and 6MWT gait speeds were significant for SCI-FAI categories 3, 4 and 5 (Wilcoxon sign rank test p<.001) (Table 5). The mean speed threshold for limited community ambulation (SCI-FAI 4) at initial evaluation was 0.39 m/s vs. 0.49 m/s for the 6MWT and 10MWT, respectively, and corresponded closely to the reported minimum speed for community ambulation of 0.44 m/s identified by van Hedel (2008)[11].

To determine if differences in gait speed between the two walk tests were functionally or clinically relevant, we calculated the MCID for all patients with enrollment and discharge measurements of the 10MWT, 6MWT, and SCI-FAI, and had SCI-FAI scores less than 5 at enrollment (n = 125). Of these patients, 78% (98/125) experienced at least a 1 unit improvement. The MCIDs were 0.11 m/s for the 6MWT and 0.15 m/s for the 10MWT (Table 4). ROC analyses showed that increases in the 6MWT and 10MWT corresponded well with 1-unit improvements in the SCI-FAI. The area under the ROC curve for the 6MWT was 0.85 (95% CI: 0.77, 0.94) and for the 10MWT was 0.83 (95% CI: 0.73, 0.92). The substantial overlap of these 95% confidence intervals indicated that the measures did not significantly differ in their correspondence with clinically relevant change. The sensitivity for each of the 6MWT and 10MWT at their respective MCID were 0.81 and 0.74 with a specificity of 0.81 for both tests.

When partitioned into slow and fast walkers, 73 slow walkers (77%) and 25 fast walkers (83%) had at least a 1 unit increase in the SCI-FAI. The MCID for slow walkers were 0.10 to 0.15; and in close correspondence with the MCID for all walkers. We did not calculate the MCID for fast walkers, because too few people were available to yield interpretable results (i.e. only 6 fast walkers failed to improve on SCI-FAI).

## Discussion

This study examined whether two common walking tests detect both statistically and clinically significant changes in walking function for a large cohort of individuals with relatively chronic iSCI. The primary finding was that walking speeds collected from the 10MWT and the 6MWT differed from each other across a broad range of speeds and for people who were self-reported in-home or community ambulators. While the speeds were correlated between the two tests, we did not find redundancy in the tests using predictive modeling. Importantly, we defined different speed thresholds for community ambulation and separate MCIDs for each test.

The current view of timed walking tests for iSCI is that the 10MWT and 6MWT produce largely equivalent measures of gait speed[4]; [5]. While strong correlations between the measures suggest redundancy, especially at slow speeds, several findings raise questions about whether outcomes measured by the 10MWT and the 6MWT are indeed equivalent. Significant differences in gait speeds collected with the 10MWT and the 6MWT have been identified in fast walkers[4]. Recent evidence also showed that the change in performance over time on these two measures for a given intervention was not strongly correlated^8^. Taken together, it appears that the 10MWT and the 6MWT may measure different aspects of walking function and is the foundation of the current study[4]; [29]–[31].

### Little Redundancy of Walking Measures for Slow and Fast Walkers

In this study, strong correlations occurred between 6MWT and 10MWT speeds for all walkers and when classified as slow or fast walkers (Table 2), which is consistent with the literature and has previously been used as evidence for redundancy between measures[4]; [9]. However, we present three lines of evidence that the 6MWT and 10MWT appear to capture different aspects of walking performance which warrant using both tests.

First, individual walking performance differed significantly as measured by the 10MWT and the 6MWT (Figure 1). Using the line of equivalence as a measure of redundancy as described by Barbeau et al.[4], we found that the line of best fit differed significantly from this for enrollment, discharge and all evaluations collected during treatment (slope<1.0). These differences remained significant for both slow and fast walkers and indicates that most slow and fast walkers had higher gait speeds on the 10MWT than on the 6MWT (Table 2). While differences have been reported for fast walkers above 0.9 m/s previously[4], this may be the first report of statistically significant differences at much slower speeds <0.44m/s but its clinical importance remains to be determined. This implies that even in slow walkers or during early recovery when walking is slow, the two tests may not capture a change in walking capacity similarly.

Second, prediction error from linear regression models can serve as another marker of redundancy. If the results from one test can be predicted with minimal error from the results of a different test, then the two tests could be considered redundant – knowledge of the results of one test provide an accurate estimate of the results of another, mitigating the need for the test being performed. The smallest real differences we calculated for the 10MWT and 6MWT (0.10 and 0.08) provide reasonable thresholds for prediction error from a linear model. Based on these thresholds, we found substantial error in the estimates of 6MWT performance at gait speeds of 0.4 m/s and above for enrollment data and 0.2 m/s and above for discharge evaluations for the entire sample.

When partitioned into slow and fast walkers, the error surpassed the SRD at a gait speed of 0.4 m/s on the 10MWT. The magnitude of the error increased as 10MWT gait speed increased, suggesting that performance on one walking test is distinct from performance on the other walking test. Importantly, the error in predicting 6MWT speeds from 10MWT speeds surpassed even the 6MWT MCID at speeds as low as 0.6 m/s. Therefore, both statistically (SRD) and clinically relevant (MCID) errors exist when predicting the performance of the 6MWT based on the 10MWT at slow, fast, and over all speeds. Therefore our data suggests that the 6MWT cannot be accurately predicted from the 10MWT via a linear regression model, in - contrast with the findings of van Hedel’s group[5]. In their study of subacute iSCI, regression analysis showed no differences between walking speeds from 6MWT and 10MWT, collected at preferred and maximum walking speeds.

The differences between our study and previous work may be explained by sample size and chronicity of the injury. Our sample was 249 subjects with time since injury ranging from 8 months to 21 years post SCI, whereas van Hedel studied a smaller sample (n = 51) and 1–6 months post SCI. In addition, in the current study, data collection occurred without physical assistance provided during ambulation, whereas earlier studies allowed up to moderate physical assistance during ambulation testing [4]. Another distinction between the reported studies and our work is the instructions given for the walking tests, in our work the instruction for 6MWT was “walk as far as you can” whereas for another study the instructions were given to, “Walk as fast as you can safely walk”[4]. Also, while differences in rehabilitation interventions surely exist, it is doubtful that the LT intervention impacted our large residual errors, because we observed these prediction errors in analyses restricted to enrollment data, before training began. The fact that residual error was similar at enrollment and discharge suggests that outcomes from the two tests reflect walking capacity more than type of intervention.

Third, differences between speeds derived from the two tests were also evident when the outcomes were classified according to function rather than a speed threshold (0.44 m/s). Using the SCI-FAI, we stratified our sample into 5 groups ranging from little or no ability to walk (SCI-FAI score 0) to some assisted or independent community ambulation (SCI-FAI score 4–5). We found statistically significant differences between 6MWT and 10MWT gait speeds for SCI-FAI classifications 3, 4 and 5 at enrollment and discharge. Our data suggest that each walking test captures walking capacity differently for slow and fast walkers and for individuals with greater capability (SCI-FAI, Table 5) and supports the use of both tests throughout recovery after an iSCI. However, if only a single test can be used, our data suggests that the 6MWT may be more responsive to walking ability. The 6MWT had a higher sensitivity than 10MWT (0.81 vs. 0.74, respectively) when measuring individuals that improved at least one category on the SCI-FAI, detecting more responders than the 10MWT. Additionally, the 6MWT had a smaller SRD than the 10MWT (Table 3). Given that SRD reflects the smallest difference needed to exceed measurement error of the test, the 6MWT appears to have less volatility than 10MWT and may be more responsive to walking recovery. Our sample is comprised of a wide range of functional abilities from walking a few steps to independent community ambulation which allows for good generalizability. In this paper, we calculated SRD with computation modifications that potentially improve the accuracy of threshold for SCI[28]; [32]; [33]. Further we calculated the value for a much larger population than has previously been reported[28]; [32]; [33]. Both of these criteria strengthen the assertion that the SRD threshold gait speed between the 6MWT and 10MWT, independent of error, is much lower than reported previously[28]. Given the lower threshold identified, previous studies might have under reported differences between these two walk tests. However SRD only peripherally relates to clinical significance since it is the value that represents change that cannot be attributed to error. Subsequently our calculated MCID values would provide much more accurate thresholds for the determination of real clinically relevant change.

### Gait Speeds Associated with SCI-FAI Functional Classifications

The 10MWT and 6MWT are often used as surrogates for functional ambulation in which gait speed over short distances (10MWT) is thought to represent crossing the street, while longer distances (6MWT) likely reflect endurance required for community ambulation. Here we directly compare gait speeds over short and long distances to self-reported walking function on the SCI-FAI mobility scale. To our knowledge, this is the first time that gait speed thresholds for different walking capacities have been determined using the 6MWT. Previously, van Hedel et al. generated 10MWT speed thresholds for 5 functional ambulation groups defined from the Spinal Cord Independence Measure (SCIM)[27]. These classifications were similar to those defined on the SCI-FAI. When considering limited community ambulation (category 4 for SCIM and SCI-FAI 4), we found that both the 6MWT and 10MWT speeds at initial and discharge aligned with van Hedel’s minimum walking speed of 0.44 m/s. These data strongly supports his assertion that 0.44 m/s is a plausible threshold for limited community ambulation after iSCI. For independent community ambulation, van Hedel proposed an average 10MWT speed of 0.80 m/s which is consistent with our 6MWT SCI-FAI Category 5 walk speed at enrollment (0.84 m/s) and discharge (0.89 m/s). However, our average 10MWT speeds were much higher than reported by van Hedel[27].

Our SCI-FAI results are novel and in contrast to Barbeau et al., who assessed walking speeds from the 15.2MWT and 6MWT in 120 subjects with iSCI and found significant separation between the two tests at gait speeds greater than 0.9 m/s[4]. We, like van Hedel et al.[11], suggest that much lower speed thresholds for community ambulation exist for iSCI regardless of whether a short or long distance walking test is used.

### Limitations

The SCI-FAI mobility subscale does have face validity, but it also demonstrates significant ceiling effects[34], as noted in the current study. Fifty-two percent of our patients identified themselves as a SCI-FAI category 5 level of mobility at enrollment which means that any improvement made during rehabilitation would not have been detected with the SCI-FAI mobility scale. These ceiling effects prevented us from calculating MCID for fast walkers. It appears that perhaps a category 6 might be warranted for the SCI-FAI, which could reflect a greater return to high-level pre-morbid ambulation activities than the current scale allows. In addition, the SCI-FAI was designed to assess functional ambulation, but walking speed is only included in the description of category 5, where speed is expected to be “at least 50% of normal”. Convergent validity of the SCI-FAI instrument was established by finding that mobility scores correlated with walking speed (Pearson r ∼ = −0.742) collected over a 3.8 meter distance, using a small sample of 22 people with incomplete SCI. The differences between our gait speed data per SCI-FAI category and the original SCI-FAI work warrants further investigation.

## Supporting Information

Appendix S1 (ZIP)
