# Supplementary material for: Are the 10 Meter and 6 Minute Walk Tests Redundant in Patients with Spinal Cord Injury?
Source: PLoS One. 2014 May 1;9(5):e94108. doi: 10.1371/journal.pone.0094108 (PMC4006773; doi:10.1371/journal.pone.0094108)
Supplement: Appendix S1 — (ZIP) [file pone.0094108.s001.zip › S1. APPENDICES. SUPPORTING INFORMATION.doc]

**APPENDIX**

*Modeling Residual Heterogeneity in Linear Models*

Standard linear models assume the homogeneity of residual variance, (i.e.) that prediction error remains constant as a function of the predictors and outcome. In our analysis, this condition stipulates that the residual variance remain constant regardless of the 10MWT speed at which we are predicting. Inspection of plots of residuals vs. fitted value (see Figure 3) provided clear evidence that the residual variance was an increase function of 10MWT speed. We thus used an extension of standard linear and mixed effects models that permit modeling the residual variance as a function of a given covariate, such as the 10MWT speed^18^. For each models we fit (at enrollment, discharge, all evaluations), we considered several different residual variance functions and conducted analysis of variance to determine which provided the best fit. In each case, the model providing the best fit to the data had residual variance increasing as a power function of the 10MWT speeds. The form of this variance function is as follows:

Residual variance = (Constant Term) * (10MWT Speed)^2*(Rate of Increase)^

The shape of this function matches the parabolic shape of the residuals plotted in Figure 3. Two parameters need to be estimated, the constant term and the rate of increase. The table below provides the estimates of these parameters for each model we fit:

| **Walk Type** | **Evaluations** | **Constant Term** | **Rate of Increase** |
| --- | --- | --- | --- |
| All Walks | Enrollment | 0.18 | 0.75 |
|  | Discharge | 0.16 | 0.41 |
|  | All Evaluations | 0.11 | 0.64 |
| Slow Walks | Enrollment | 0.17 | 0.76 |
|  | Discharge | 0.26 | 0.91 |
|  | All Evaluations | 0.06 | 0.30 |
| Fast Walks | Enrollment | 0.16 | 0.58 |
|  | Discharge | 0.16 | 0.11 |
|  | All Evaluations | 0.11 | 0.56 |

*Modification of Smallest Real Difference Calculation*

Beckerman et al.^21^ suggests the formula SRD = SD * √2 * √(1 – r) * 1.96, where SD represents the standard deviation of a set of measurements of an outcome and r represents the intraclass correlation coefficient. The rationale for this number is that it is a constant multiple of the square root of the proportion of the variance attributable to measurement error used in a two-group z-test – the SD * √2 * √(1 – r) represents the square root of the proportion of the two-group z-test variance and 1.96 is the 97.5% quantile of the standard normal distribution (for a two-tailed test at significance level .05). This formulation, however, ignores the fact that the appropriate test of effectiveness of an intervention is a paired z-test (or other alternative paired test such as the t- or Wilcoxon signed rank tests), and that the appropriate variance to use is the variance of the difference between pre- and post-intervention measurements. Even under the assumption of equal variances for the pre- and post-intervention measurements, the variance of the differences is twice the variance of the pre-intervention measurements less the covariance of the pre- and post-intervention measurements. This covariance is rarely null, given that pre- and post-intervention measurements taken on a subject are generally correlated. A more appropriate formula for the SRD would remove the √2 term and replace the standard deviation of a set of measurements on an independent subject with the standard deviation of the difference between pre- and post-intervention measurements, (i.e.) SRD = SD * √(1 – r) * 1.96, where SD represents said standard deviation.
